# Intérêt du score d’Alvarado dans le diagnostic des appendicites aigües

**DOI:** 10.11604/pamj.2018.29.56.14011

**Published:** 2018-01-22

**Authors:** Houcine Maghrebi, Hamida Maghraoui, Amine Makni, Amine Sebei, Sabri Ben Fredj, Ali Mrabet, Kamel Majed, Nabiha Falfoul, Zoubeir Bensafta

**Affiliations:** 1 Service de Chirurgie A, Hôpital La Rabta, Tunis, Tunisie; 2 Service des Urgences, Hôpital La Rabta, Tunis, Tunisie; 3 Faculté de Médecine de Tunis, Tunisie

**Keywords:** Urgences, appendicite aigüe, score, chirurgie générale, Emergencies, acute appendicitis, score, general surgery

## Abstract

**Introduction:**

L’appendicite aigue représente l’urgence chirurgicale la plus fréquente aux urgences. Son diagnostic est avant tout clinique. Cependant, sa présentation clinique parfois trompeuse ainsi que le large éventail de diagnostics différentiels sont fréquemment sources d’erreurs diagnostiques et de retard de prise en charge. Afin de pallier à ces difficultés diagnostiques, de réduire le nombre d’examens complémentaires et d’actes chirurgicaux abusifs, plusieurs scores cliniques ont été ainsi développés, dont le score d’Alvarado. L’objectif de cette étude était d’appliquer ce score à une population de patients adultes venant consulter pour douleurs de la fosse iliaque droite afin d’évaluer ses performances ainsi que ses limites.

**Méthodes:**

Il s’agissait d’une étude prospective qui a inclus tous les malades âgés de plus de 15 ans se présentant pour douleur de la fosse iliaque droite. Le diagnostic final d'appendicite aigue a été confirmé par examen anatomo-pathologique pour les patients opérés et infirmé lorsque la symptomatologie des patients avait totalement régressé en l'absence de tout traitement.

**Résultats:**

Notre étude a inclus 106 patients. Chez les patients dont le score d'Alvarado était inférieur à 4, le diagnostic d'appendicite aigüe n'a jamais été retenu. Les meilleures sensibilités et spécificités ont été retrouvées pour une valeur seuil de 8 pour le score d'Alvarado. Ainsi, avec une bonne sensibilité (81,25%) et une valeur prédictive positive correcte (74,28%), notre étude a démontré que le score d’Alvarado pouvait apporter un bénéfice dans le diagnostic d'appendicite aigüe. Le groupe de patient avec un score strictement inférieur à 4 est considéré comme à faible risque. Les patients dont le score d'Alvarado était strictement supérieur à 6 nécessiteraient une hospitalisation afin, soit d'être opérer d'emblée, soit de compléter par un examen d'imagerie et une surveillance. Le groupe de patient ayant un score compris entre 4 et 6 (limites incluses), reste un groupe où le doute diagnostic est présent et où les examens complémentaires d'imagerie ont un apport certain.

**Conclusion:**

L’utilisation du score d'Alvarado dans nos urgences permet de rationaliser la prise en charge et d’orienter le diagnostic en limitant la prescription d’explorations radiologiques, le coût de la prise en charge et les actes chirurgicaux abusifs.

## Abstract (en)

**Introduction:**

Acute appendicitis is the most frequent surgical emergency in the Emergency Department and diagnosis is mainly based on clinical examination. However, its clinical features are sometimes deceptive as well as the wide range of differential diagnoses are frequently sources of diagnostic errors and of a delayed management. In order to reduce these diagnostic difficulties, the number of complementary examinations and abusive surgical procedures, several clinical scores have been developed, including the Alvarado score. The objective of this study aimed to apply this score to a population of adult patients presenting with pain in the right iliac fossa in order to evaluate its performance as well as its limits.

**Methods:**

We conducted a prospective study of all patients over the age of 15 years presenting with pain of the right iliac fossa. In patients undergoing surgery, the final diagnosis of acute appendicitis was confirmed by the anatomo-pathological examination while it was negated when the symptoms regressed in the absence of any treatment.

**Results:**

Our study included 106 patients. In patients with Alvarado score less than 4, the diagnosis of acute appendicitis was never retained. The best sensitivities and specificities were found in patients with a threshold value of 8 (Alvarado score). Our study demonstrated that Alvarado score can provide immediate benefit in the diagnosis of acute appendicitis based on its good sensitivity (81.25%) and correct positive predictive value (74,28%). The group of patients with a score strictly less than 4 was considered to be low risk. The patients with Alvarado score strictly greater than 6 would require hospitalization either to undergo emergency surgery or to undergo imaging examinations and be monitored. In the group of patients with a score between 4 and 6 (extremes included) there was no diagnostic certainty, hence complementary imaging techniques were essential.

**Conclusion:**

In our Emergency Department Alvarado score helps clinicians to streamline patient’s management and to give an indication to the diagnosis by limiting the prescription of radiological examinations, the cost of patients’ management and the abusive surgical procedures.

## Introduction

L’appendicite aigue représente l’urgence chirurgicale la plus fréquente aux urgences avec un risque de la développer durant la vie oscillant entre 6 et 9% [1,2]. Son diagnostic est avant tout clinique. En effet, devant un tableau typique, l’intervention chirurgicale s’impose, sans aucune investigation complémentaire préalable. Cependant, sa présentation clinique très variée et le large éventail de diagnostics différentiels sont sources d’erreurs diagnostiques et de retard de prise en charge, même pour les cliniciens les plus expérimentés. Ces difficultés diagnostiques (30 à 50% des cas) peuvent, soit induire des retards de prise en charge, soit motiver la réalisation d’une exploration chirurgicale, pouvant s’avérer « blanche » [1]. Afin de pallier à ces difficultés diagnostiques, mais aussi dans le but de réduire le nombre d’examens complémentaires et d’actes chirurgicaux abusifs, des études ont tenté de mieux définir les indications opératoires par l’établissement de scores clinico-biologiques prédictifs d’appendicite aigue. Plusieurs scores cliniques ont ainsi été développés en particulier le score d’Alvarado qui est le plus connu et le plus diffusé dans la littérature médicale [3]. L’objectif de cette étude était d’appliquer ce score à une population de patients adultes venant consulter pour douleurs de la fosse iliaque droite afin d’évaluer ses performances ainsi que ses limites.

## Méthodes

**Type d’étude:** Il s’agissait d’une étude unicentrique, prospective, observationnelle.

**Lieux et durée de l’étude:** Notre travail a été mené aux urgences de l'hôpital la Rabta sur une période étendue du 25 Décembre 2014 au 24 Février 2015.

### Critères de sélection des participants

**Critères d’inclusion:** On a inclu tous les malades de plus de 15 ans se présentant aux urgences pour des douleurs de la fosse iliaque droite.

**Critères de non inclusion:** N'ont pas été inclus les patients âgés de moins de 15 ans, ceux ayant des antécédents d'appendicectomie et les femmes enceintes.

**Critères d’exclusion:** Ont été exclus de l'étude les patients dont les données pour calculer le score d'Alvarado étaient manquantes, les patients perdus de vue lors du suivi et les patients ayant retiré leur consentement pour la participation à l'étude.

**Procédure de collecte:** Le recueil des données a été réalisé par le médecin de garde au niveau du box des urgences chirurgicales. Outre les données démographiques, les différents items du score d’Alvarado ont été relevés et le score a été calculé pour chaque patient. La décision d’opérer ou de réaliser des examens complémentaires a été prise par le chirurgien de garde, indépendamment du score d’Alvarado calculé par le médecin expérimentateur. Concernant les patients hospitalisés, les données de suivi du dossier d'hospitalisation ont été consignées. Les patients non opérés ont été convoqués après 15 jours. Le diagnostic final d'appendicite aigue a été confirmé par l’examen anatomo-pathologique pour les patients opérés.

Le calcul du score d’Alvarado a été réalisé comme l’indique la littérature en tenant compte des six critères cliniques et des deux critères biologiques, pondérés par des coefficients selon une grille de calcul (Tableau 1). Les patients ont été par la suite répartis en 3 groupes en fonction de leur score d'Alvarado: le Groupe A comprenait les patients avec un score d’Alvarado strictement inférieur à 4. Ceux dont le score était compris entre 4 et 6 représentaient le groupe B. Et enfin ceux dont le score était strictement supérieur à 6 représentaient le groupe C.

**Tableau 1:** Calcul du score d’Alvarado

Items du score	Score
Douleur avec migration	1
Anorexie	1
Nausées et/ou vomissements	1
Sensibilité fosse iliaque droite	2
Température supérieure à 37,3°C	1
Décompression de la fosse iliaque gauche douloureuse	1
Taux de leucocytes supérieur à 10 000/ml	2
Décalage des polynucléaires neutrophiles à gauche	1
Total	10

### Analyse des données

Nous avons calculé des fréquences simples et des fréquences relatives (pourcentages) pour les variables qualitatives. Nous avons calculé des moyennes, des médianes et des écarts-types et déterminé les valeurs extrêmes pour les variables quantitatives. Les comparaisons de 2 moyennes sur séries indépendantes ont été effectuées au moyen du test t de Student pour séries indépendantes. Les comparaisons de pourcentage sur séries indépendantes ont été effectuées par le test du chi-deux de Pearson et en cas de non-validité de ce test, et de comparaison de 2 pourcentages, par le test exact bilatéral de Fisher.

Pour la détermination du seuil auquel il faut « couper » la variable quantitative, nous avons établi des courbes ROC (Receiver Operating Characteristics). Après avoir vérifié que l’aire sous la courbe est significativement > 0,500, nous avons choisi comme seuil la valeur de la variable qui correspond au meilleur couple « sensibilité-spécificité ». Dans tous les tests statistiques, le seuil de signification a été fixé à 0,05.

**Aspects éthiques:** L'étude était observationnelle descriptive, respectant l’autonomie de l’équipe et n’a imposé aucune exploration supplémentaire.

## Résultats

**Figure 1:**
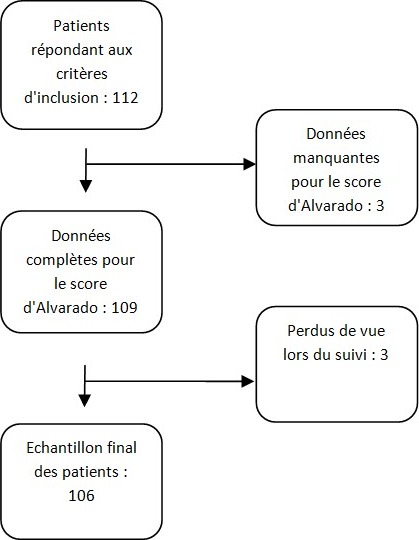
Flow chart de l’étude

Nous avons inclus 106 patients répondant aux critères d’inclusion (Figure 1). L’âge moyen des patients recrutés était de 30 ans (15, 70 ans). La tranche d’âge (20 - 29 ans) représentait 43,4% des cas. Le sexe ratio H/F était de 0,86. Le score d'Alvarado a été calculé pour tous les patients. La médiane était de 7, avec un écart type à 1,98. Les valeurs allaient de 2 à 10. Les différentes valeurs du score d'Alvarado avec les correspondances avec ou sans geste opératoire sont résumées dans le Tableau 2. Chez les patients dont le score d'Alvarado était inférieur à 4, le diagnostic d'appendicite aigüe n'a jamais été retenu. Les correspondances entre le score d'Alvarado et le diagnostic d'appendicite aigüe sont résumées dans le Tableau 3. Les meilleures sensibilités et spécificités ont été retrouvées pour une valeur seuil de 8 pour le score d'Alvarado. Ainsi, un score d'Alvarado supérieur ou égal à 8 multiplie la probabilité d'appendicite aigue par 9,5 (p < 0,0001). Les patients ont été par la suite répartis en 3 groupes en fonction de leur score d'Alvarado. Les résultats sont rapportés dans le Tableau 4. Un score d'Alvarado inférieur ou égal à 6 était significativement lié à l'absence d'appendicite aigüe (Tableau 5). Ainsi un score d'Alvarado supérieur à 6 multipliait la probabilité de diagnostic d'appendicite aigue par 5,7. La sensibilité obtenue par cette répartition s'élevait à 81,3%. La spécificité était à 57%. La VPP était de 74,3%. La VPN était de 66,6%. Par ailleurs, chez les patients avec un score d'Alvarado inférieur à 4, les échographies abdominales n'avaient pas objectivé d'appendicite aigüe, dans les limites de l'examen. Chez les patients avec un score d'Alvarado inférieur à 4, la TDM abdominale n'avait pas objectivé d'appendicite aigüe. Toutes les appendicites à la TDM ont été ensuite confirmées par l'examen anatomo-pathologique.

**Tableau 2:** Répartition des patients en fonction du score et des pourcentages d'opérés

Score d'Alvarado	Opération	
	Oui	Non
< à 4	0 (0%)	6 (16,2%)
[4 - 6]	14 (20,3%)	16 (43,2%)
> à 6	55 (79,7%)	15 (40,5%)

**Tableau 3:** Appendicite aigüe en fonction du score d'Alvarado

Score d'Alvarado	Appendicite N = 64	Appendice sain N = 42
< à 4	0 (0%)	6 (14,2%)
[4 - 6]	12 (18,8%)	18 (42,9%)
> à 6	52 (81,2%)	18 (42,9%)

**Tableau 4:** Répartition des patients en fonction du score d'Alvarado et du diagnostic final

Score d'Alvarado	Appendicite N = 64	Appendice sain N = 42	P du groupe
< à 4	0 (0%)	6 (14,2%)	0,003
[4 - 6]	12 (18,8%)	18 (42,9%)	0,007
> à 6	52 (81,2%)	18 (42,9%)	0,0001

**Tableau 5:** Répartition des patients par rapport à la valeur seuil de 6 du score d'Alvarado et étude de corrélation

Score d'Alvarado	Appendicite	Appendice sain	P	Odds ratio	IC à 95%
≤ à 6	12 (18,8%)	24 (57,1%)	**0,00004**	**5,7**	**2,22-15,35**
> à 6	52 (81,2%)	18 (42,9%)			

## Discussion

Notre étude a montré que l'utilisation du score d'Alvarado dans nos urgences pour les suspicions d'appendicite aigüe permettrait d'orienter le diagnostic, tout en évitant la prescription d’explorations radiologiques inutiles et de limiter par conséquent les délais d’attente aux urgences, ainsi que le coût de la prise en charge. En effet, malgré que l’appendicite aigue représente l’urgence chirurgicale la plus fréquente aux urgences, le chirurgien dans sa pratique courante peut se retrouver face à une situation de doute diagnostic avec parfois un recours difficiles aux examens complémentaires d'imagerie. Dans ces situations, le score d'Alvarado peut être utilisé comme critère objectif d'admission. Dans notre étude, pour les scores inférieurs à 4, le diagnostic d'appendicite semble peu probable et les patients pourraient être renvoyés à leur domicile sans exploration supplémentaire. Pour les scores situés entre 4 et 6, bien qu'ils aient été associés à un risque faible de diagnostic positif d'appendicite aigüe dans notre étude, il est recommandé, particulièrement chez la femme, de compléter par un examen morphologique. Un score supérieur à 6 devrait indiquer l'hospitalisation en vue d'une surveillance rapprochée ou d'un geste chirurgical immédiat.

Chan, dans une étude prospective portant sur 175 patients [4], propose un algorithme basé sur le score d'Alvarado pour sélectionner les patients à admettre pour surveillance. Tout patient présentant un score d'Alvarado supérieur à 4 est à hospitaliser pour surveillance ou examen complémentaire. Aucun patient avec un score inférieur à 4 n'avait présenté d'appendicite aigüe. Cette conduite nous parait reproductible selon les résultats de notre étude, en y ajoutant l'apport d'un examen d'imagerie pour les scores compris entre 4 et 6. Dans un protocole prospectif, Winn et al [5] ont proposé une attitude thérapeutique en fonction du score calculé. Dans une étude prospective unicentrique publiée au Journal de Chirurgie Viscérale en 2010, Pouget [6] avait inclus 233 patients adultes se présentant aux urgences pour douleur de la fosse iliaque droite. L’analyse des résultats montrait qu’un score inférieur à 4 était significativement lié à l’absence d’appendicite aiguë et un score supérieur à 6 était significativement lié à une appendicite aiguë à traiter chirurgicalement. Mais cette étude ne permettait pas de conclure pour un score compris entre 4 et 6. La VPP retrouvée dans cette étude était significativement plus élevée que dans notre étude pour la valeur du score d'Alvarado supérieur à 6, soit 89,2%. De plus, la sensibilité retrouvée dans cette même étude était là aussi plus élevée, à 92,8%. Ces différences pourraient s'expliquer par une différence du nombre de patients inclus dans l'étude par rapport à celle de Pouget. Il en est de même pour l'étude de Limpawattanisiri [7] où l'effectif était de 1.000 patients. Enfin, l’équipe de Robert Ohle a publié en 2011 dans le journal BMC Medicine, une revue de la littérature traitant du score d’Alvarado [8]. Ils ont recherché dans Medline, Embase, DARE et la bibliothèque Cochrane toutes les études de validation du score. Quarante-deux études ont été incluses. Un cut off à 5 était suffisant pour exclure le diagnostic d’appendicite (sensibilité de 99%). Le score d'Alvarado était considéré comme un moyen sure et efficace essentiellement chez les adultes de sexe masculin. Cependant une surestimation de la probabilité d'appendicite à noter chez les femmes.

Avec une bonne sensibilité (81,25%) et une valeur prédictive positive correcte (74,28%), notre étude a démontré que le score d’Alvarado pouvait apporter un bénéfice dans le diagnostic d'appendicite aigüe. Pour un score strictement inférieur à 4, aucun patient n'a souffert d'appendicite aigüe. Ce groupe est considéré comme à faible risque. Les patients dont le score d'Alvarado était strictement supérieur à 6 nécessiteraient une hospitalisation afin soit d'être opérer d'emblée, soit de compléter les explorations par un examen d'imagerie ou d’une surveillance. Le groupe de patient ayant un score compris entre 4 et 6 (limites incluses) reste un groupe où le doute diagnostic est présent, et où les examens complémentaires d'imagerie ont un apport certain. L'apport du score était entre autres la rationalisation des demandes d'examens radiologiques complémentaire chez les patients se présentant pour douleur de la fosse iliaque droite. En effet, chez les patients avec un score d'Alvarado inférieur à 4, les échographies abdominales n'avaient pas objectivé d'appendicite aigüe, dans les limites de l'examen. De même, la TDM abdominale n'avait pas objectivé d'appendicite aigüe chez les patients avec un score d'Alvarado inférieur à 4.

## Conclusion

La prise en charge classique de l'appendicite aigüe doit se baser sur un interrogatoire minutieux à la recherche des tares présentées par le patient, de la symptomatologie fonctionnelle, ainsi qu'un examen clinique minutieux et répété. Le bon sens clinique de l'urgentiste et l'expérience du chirurgien devraient toujours primer dans la prise en charge de ces patients, dans le but de ne pas la retarder. Toutefois, l’apport des différents scores d’aide au diagnostic est certain, la population de jeunes médecins devrait en profiter pour rationaliser la prise en charge, orienter le diagnostic en limitant la prescription d’explorations radiologiques, le coût de la prise en charge et les actes chirurgicaux abusifs.

### Etat des connaissances actuelles sur le sujet

- L’appendicite aigue représente l’urgence chirurgicale la plus fréquente;
- Sa présentation clinique parfois trompeuse ainsi que le large éventail de diagnostics différentiels sont fréquemment sources d’erreurs diagnostiques et de retard de prise en charge;
- Ces difficultés diagnostiques (30 à 50% des cas) peuvent, soit induire des retards de prise en charge, soit motiver la réalisation d’une exploration chirurgicale, pouvant s’avérer « blanche ».

### Contribution de notre étude à la connaissance

- L’utilisation du score d'Alvarado dans nos urgences pour les suspicions d'appendicite aigüe permettrait d'orienter le diagnostic, tout en évitant la prescription d’explorations radiologiques inutiles et de limiter par conséquent les délais d’attente aux urgences, ainsi que le cout de la prise en charge;
- Ainsi un score d'Alvarado supérieur à 6 multipliait la probabilité de diagnostic d'appendicite aigue par 5,7;
- Un score d'Alvarado inférieur ou égal à 6 était significativement lié à l'absence d'appendicite aigüe.

## Conflits d’intérêts

Les auteurs ne déclarent aucun conflit d’intérêts.
